# SARS-CoV-2 strains bearing Omicron BA.1 spike replicate in C57BL/6 mice

**DOI:** 10.3389/fimmu.2024.1383612

**Published:** 2024-04-29

**Authors:** Patricia P. Ogger, Minerva Garcia Martín, Soyeon Jang, Jie Zhou, Jonathan Brown, Ksenia Sukhova, Wilhelm Furnon, Arvind H. Patel, Vanessa Cowton, Massimo Palmarini, Wendy S. Barclay, Cecilia Johansson

**Affiliations:** ^1^ Section of Respiratory Infections, National Heart and Lung Institute, Imperial College London, London, United Kingdom; ^2^ Department of Infectious Disease, Imperial College London, London, United Kingdom; ^3^ MRC-University of Glasgow Centre for Virus Research, Glasgow, United Kingdom

**Keywords:** SARS-CoV-2, variant, respiratory infection, spike protein, knockout mice

## Abstract

**Introduction:**

SARS-CoV-2, the cause of the COVID pandemic, is an RNA virus with a high propensity to mutate. Successive virus variants, including variants of concern (VOC), have emerged with increased transmission or immune escape. The original pandemic virus and early variants replicated poorly, if at all, in mice at least partly due to a mismatch between the receptor binding domain on the viral spike protein and the murine angiotensin converting enzyme 2 (ACE2). Omicron VOC emerged in late 2021 harboring > 50 new mutations, 35 of them in the spike protein. This variant resulted in a very large wave of infections, even in the face of prior immunity, albeit being inherently less severe than earlier variants. Reflecting the lower severity reported in humans, Omicron displayed attenuated infection in hamsters and also in the K18-*hACE2* mouse model. K18-*hACE2* mice express both the human ACE2 as well as the endogenous mouse ACE2.

**Methods:**

Here we infected *hACE2* ^knock-in^ mice that express only human ACE2 and no murine ACE2, or C57BL/6 wildtype mice with SARS-CoV-2 D614G (first-wave isolate), Delta or Omicron BA.1 variants and assessed infectivity and downstream innate immune responses.

**Results:**

While replication of SARS-CoV-2 Omicron was lower in the lungs of *hACE2* ^knock-in^ mice compared with SARS-CoV-2 D614G and VOC Delta, it replicated more efficiently than the earlier variants in C57BL/6 wildtype mice. This opens the opportunity to test the effect of host genetics on SARS-CoV-2 infections in wildtype mice. As a proof of principle, we tested Omicron infection in mice lacking expression of the interferon-alpha receptor-1 (IFNAR1). In these mice we found that loss of type I IFN receptor signaling resulted in higher viral loads in the lungs were detected. Finally, using a chimeric virus of first wave SARS-CoV-2 harboring the Omicron spike protein, we show that Omicron spike increase infection of C57BL/6 wildtype mice, but non-spike genes of Omicron confer attenuation of viral replication.

**Discussion:**

Since this chimeric virus efficiently infected C57BL/6 wildtype mice, and replicated in their lungs, our findings illustrate a pathway for genetic mapping of virushost interactions during SARS-CoV-2 infection.

## Introduction

SARS-CoV-2 emerged from an animal source in late 2019 and caused the COVID pandemic. After approximately 1 year of human circulation, new viruses evolved, including variants of concern (VOC), with hallmarks of increased transmissibility, disease severity, or reduced vaccine efficacy and immune evasion (1). Major worldwide waves in 2021 were attributed to the Alpha and Delta VOCs (2, 3). In November 2021, Omicron VOC was detected in South Africa and Botswana, harboring >50 new mutations with 35 mutations in the spike gene (4), initiating a new global infection wave.

The majority of the mutations that define successive variants, including Omicron, have occurred within the spike (S) gene. Spike protein, which binds to the receptor ACE2 with its receptor-binding domain (RBD) (5), is critical for the fusion of the virus and cell membranes (6) during cell entry. S is the major target for antibodies that neutralize viruses by blocking receptor binding or preventing fusion (7). Here we compared the replication of first-wave SARS-CoV-2 with Delta and Omicron BA.1 VOCs in several mouse models. In Delta VOC, S mutations are known to enhance syncytium formation, a phenotype associated with pathogenesis (8). Although binding of the Omicron spike to the ACE2 receptor remains similar to Delta (9), Omicron lacks fusogenicity, and this may at least partly account for its decreased severity (10, 11). In contrast to Delta and all earlier VOCs where viral membrane fusion and cell entry may occur at the cell surface, Omicron can enter cells via the endosomal route (11). This phenotype has been mapped to mutations in the spike protein S2 domain (12).

SARS-CoV-2 VOCs have been studied in animal models to better understand infection dynamics, time frames, and downstream immune responses (13). First-wave and early VOCs were poorly infectious in wildtype mice, at least partly because the spike protein did not bind to the murine ACE2. *hACE2* ^knock-in^ mice (14) overcome this limitation while at the same time abrogating data convolution by the presence of mouse ACE2, which could allow cell entry in some, but not all, variants. Some of the mutations in the Omicron spike protein facilitate interaction with mouse ACE2, enabling infection of mice not expressing hACE2 (15). Interestingly, Omicron infection is still attenuated in both K18-*hACE2* and wild-type (WT) 129 mice (16). While this may be partly attributed to spike protein mutations that impact cell entry pathways, further investigation showed that mutations outside the spike protein, such as a three-amino-acid deletion in non-structural protein 6 (Nsp6), which contributes to the formation of replication organelles, contribute to lower viral loads (15, 17). However, it remained unclear whether the spike protein of Omicron BA.1 results in attenuated disease in other mouse strains such as the *hACE2* ^knock-in^ strain, which, as opposed to K18-*hACE2* mice, replaces the murine with the human ACE2 (14), and whether or not Omicron BA.1 could infect the widely used C57BL/6 WT strain, which is most often the genetic background of genetically altered mice.

Here we infected *hACE2* ^knock-in^ and C57BL/6 WT mice with SARS-CoV-2 D614G (first-wave isolate), Delta or Omicron BA.1 variants and studied infectivity and downstream innate immune responses. This showed that SARS-CoV-2 Omicron BA.1 could infect C57BL/6 WT mice, resulting in high viral loads and induction of antiviral effectors and cytokines. However, in *hACE2* ^knock-in^ mice, SARS-CoV-2 Omicron BA.1 was poorly infectious and did not induce innate immune responses. The infectivity of SARS-CoV-2 Omicron BA.1 in mice lacking the hACE2 receptor opened the possibility to investigate the impact of other host factors on the infection of SARS-CoV-2 such as the lack of type I IFN responses using the interferon-alpha receptor-1 (IFNAR1) knockout mice. Finally, using a chimeric virus of SARS-CoV-2 D614G harboring the Omicron BA.1 spike protein, we show that indeed it is the Omicron BA.1 spike that drives virus entry of SARS-CoV-2 Omicron BA.1 in WT mice, and combined with the rest of the viral genes from first-wave virus D614G, this drives increased viral load and innate immune responses in mice. Therefore, this offers a robust small animal model for genetic mapping of SARS-CoV-2 virus–host interactions.

## Materials and methods

### Biosafety and ethics

All experiments were approved by the local genetic manipulation safety committee of Imperial College London, St. Mary’s Campus (center number GM77) and the Health and Safety Executive of the United Kingdom, under reference CBA1.77.20.1. SARS-CoV-2 reverse genetics was performed at CVR University of Glasgow.

### Mice

*hACE2* ^knock-in^ mice were purchased from The Jackson Laboratory (Line 035000) and bred in-house at Imperial College London. All animal studies were reviewed and approved by the Animal Welfare and Ethical Review Board (AWERB) at Imperial College London and approved by the UK Home Office in accordance with the Animals Act 1986 (Scientific Procedures) and ARRIVE guidelines.

### Virus and infections

First-wave SARS-CoV-2 (D614G, isolate of hCoV-19/England/IC19/2020), Delta (B.1.617.2, isolate of hCoV-19/England/SHEF-10E8F3B/2021), and Omicron (Omicron/BA.1, isolate of hCoV-19/England/PHEP-YYNTNOA/2021) were grown in African green monkey kidney cells overexpressing human ACE2 and TMPRSS2 (Vero-ACE2-TMPRSS2; VAT cells) (18). Reverse-genetics-derived viruses were generated at the CVR as previously described (11, 19, 20). Recombinant SARS-CoV-2 Wuhan cDNA genomes bearing the Omicron BA.1 spike-encoding sequence were assembled using the transformation-associated recombination (TAR) method as described previously (11). These genomes were used as a template to *in vitro*-transcribed viral genomic RNA and subsequently transfected into BHK-*hACE2*-N cells, which stably express SARS-CoV-2 N and hACE2 for virus rescue. The rescued viruses were passaged once in Vero E6 cells and sequenced using Oxford Nanopore to confirm their identity.

For infection, mice were lightly anesthetized and instilled intranasally (i.n.) with 2 × 10^5^ plaque-forming units (PFU) of SARS-CoV-2 or phosphate-buffered saline (PBS) in 100 µl.

### Plaque assays

SARS-CoV-2 titer was assessed in lungs at 0.75, 2, and 7 d.p.i. using a plaque assay. In brief, serial dilutions of lung homogenate in serum-free Dulbecco’s modified Eagle medium [DMEM, containing 1% non-essential amnio acids (NEAA), 100 U/mL penicillin, and 100 µg/mL streptomycin) were performed and inoculated onto VAT cells for 1 h at 37°C. The inoculum was then removed and replaced with overlay medium (1× MEM, 0.2% w/v BSA, 0.16% w/v NaHCO_3_, 10 mM HEPES, 2 mM L-glutamine, 100 U/mL penicillin, 100 µg/mL streptomycin, and 0.84% agarose). Plates were incubated for 3 days at 37°C before the overlay was removed, and cells were stained for 1 h at room temperature in 0.5% crystal violet solution. The virus plaques were counted and multiplied by the dilution factor to calculate titer as plaque-forming unit/mL (PFU/mL).

### Isolation of lung cells

Mice were sacrificed at 0.75, 2, and 7 d.p.i., and lungs were perfused with PBS. To obtain lung leukocytes, lung lobes were cut into smaller pieces and incubated in complete DMEM (cDMEM, supplemented with 10% fetal bovine serum, 2 mM L-glutamine, 100 U/mL penicillin, and 100 µg/mL streptomycin), 1 mg/mL Collagenase D (Roche), and 30 µg/mL DNase I (Invitrogen) for 1 h at 37°C and then mashed through a 100-µm filter (BD). Red blood cells were lysed using ammonium chloride potassium buffer.

### BAL cell processing

BAL was collected by flushing the lungs three times with 1 mL PBS supplemented with 5 mM EDTA (Life Technologies). The BAL cells and supernatant were separated by centrifugation, and the BAL supernatants were exposed to UV light for 2 min to inactivate SARS-CoV-2. Red blood cells were lysed using ammonium chloride potassium buffer.

### RNA isolation and quantitative RT-PCR

Lung tissue was homogenized in TRIzol, and RNA extraction was performed according to the manufacturer’s instructions. After the chloroform step, the aqueous phase containing RNA was further processed using RNeasy Mini Kit (QIAGEN) according to the manufacturer’s instructions. Furthermore, 2 µg RNA was reverse-transcribed using a high-capacity RNA-to-cDNA kit (Applied Biosystems) according to the manufacturer’s instructions. To quantify the mRNA levels in lung tissue, quantitative RT-PCR was performed using QuantiTect Probe PCR Master Mix (Qiagen) and 7500 Fast Real-Time PCR System (Applied Biosystems). For *Ifna*, *Cxcl1*, *Cxcl10*, *Mx1*, *Ccl2*, and *Il6* (all primer–probe mixes from Applied Biosystems) and SARS-CoV-2, *N* and *E* gene (21) expression was calculated relative to the expression of *Gapdh*. First, the ΔCT (CT = cycle threshold) between the target gene and *Gapdh* was calculated for each sample, followed by the calculation of 2^-ΔCT^. The gene expression for *Oas1*, *Viperin*, *Ifnb*, and *Ifnl* was performed using primers and probes as previously described (22). For the absolute quantification of these genes, the exact number of copies of the genes was calculated using a plasmid DNA standard curve, and the results were normalized to the levels of *Gapdh* (Applied Biosystems).

### Statistical analysis

Statistical analysis was performed using Prism 6 (GraphPad Software). One-way ANOVA with Tukey’s *post-hoc* test was used to compare multiple groups. Data are expressed as mean ± SEM, and for all tests, a value of *P <*0.05 was considered significant (*p < 0.05, **p < 0.01, ***p < 0.005, ****p < 0.001).

## Results

### Infection with SARS-CoV-2 variants results in different degrees of viral load and ISG response in *hACE2* ^knock-in^ mice

**Figure 1:**
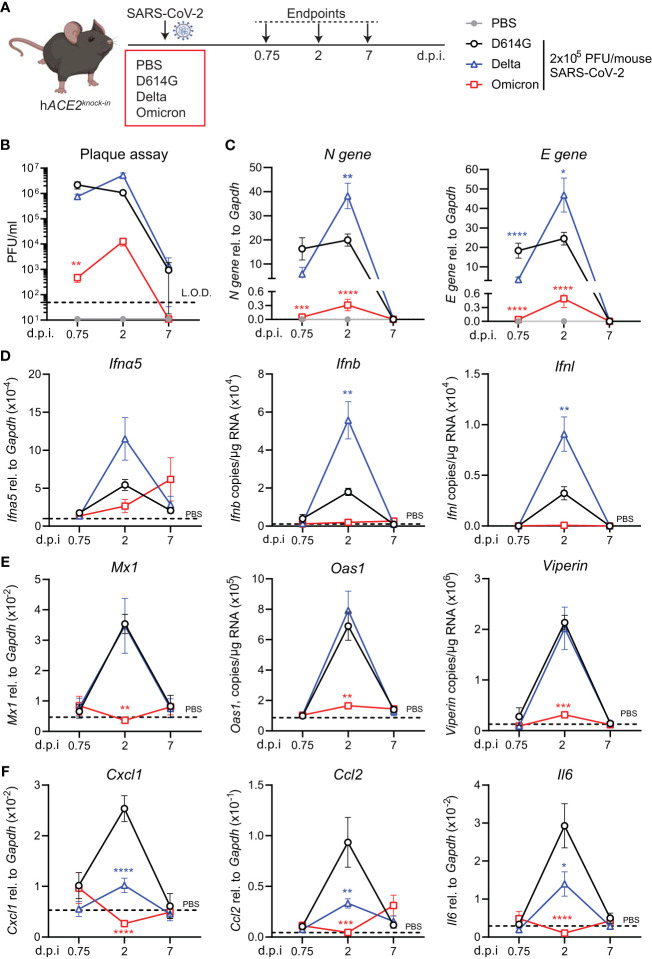
Viral replication and ISG response after infection with different variants of SARS-CoV-2 in the *hACE2^knock-in^* model. **(A)** *hACE2^knock-in^* mice were intranasally infected with 2 × 10^5^ PFU SARS-CoV-2 D614G, Delta (B.1.617.2) or Omicron (B.1.1.529) variants, or mock (PBS). Lungs and BAL were harvested at 0.75, 2, and 7 days post-infection (d.p.i). **(B)** Viral load measured by plaque assay on Vero cells overexpressing hACE2 and TMPRESS2, limit of detection (L.O.D. = 50 PFU/mL). **(C)** Expression of SARS-CoV-2 *N gene* (nucleocapsid phosphoprotein) and *E gene* (envelope protein) in lung tissue relative to *Gapdh*, measured by RT-PCR. **(D, E)** Gene expression analysis of type I and III; IFNs *Ifna5, Ifnb*, and *Ifnl* **(D)** and ISGs; *Mx1*, *Oas1*, and *Viperin* **(E)** measured by RT-PCR shown as total copy number normalized to the expression of *Gapdh* or relative to the expression of *Ifna5* and *Gapdh* (*Mx1*). **(F)** Gene expression of *Cxcl1, Ccl2*, and *Il6*, measured by RT-PCR relative to the expression of *Gapdh.* Data are shown as mean ± SEM; two experiments pooled, *n* = 6–8 per group. PBS control *n* = 11, shown in **(D–F)** as dotted line. One-way ANOVA with Tukey’s multiple-comparison test per time point was used; * indicates significance compared to SARS-CoV-2 WT-infected group; *p < 0.05, **p < 0.01, ***p < 0.005, ****p < 0.001.

To investigate virus replication and interferon-stimulated gene (ISG) induction in *hACE2* ^knock-in^ mice upon infection with SARS-CoV-2 Delta or Omicron (BA.1) variants compared to first-wave SARS-CoV-2 (D614G), mice were infected by inoculation with 2 × 10^5^ plaque-forming units (PFU) of each SARS-CoV-2 variant or mock infected with PBS. Lungs were harvested at days 0.75, 2, and 7 post-infection (p.i.) and homogenized (**Figure 1A**). As reported for other mouse strains (16, 23), infection with SARS-CoV-2 D614G, Delta, or BA.1 did not induce any weight loss in *hACE2* ^knock-in^ mice (**Supplementary Figure S1A**). Plaque assays on Vero cells overexpressing ACE2 and transmembrane protease serine 2 precursor TMPRSS2 (VAT cells) revealed high titers, 10^6^ PFU or more, of infectious virus in the lungs of SARS-CoV-2 D614G and Delta VOC-infected mice on days 0.75 and 2 p.i. that were not completely cleared by day 7 p.i. (**Figure 1B**). In contrast, early infectious viral loads were significantly lower in the lungs of mice infected with SARS-CoV-2 Omicron BA.1 and below the detection limit at day 7 p.i. (**Figure 1B**). This pattern was confirmed by the gene expression analysis of SARS-CoV-2 nucleocapsid (N) and envelope (E) genes. While N and E gene expression was detected in all SARS-CoV-2-infected groups (**Figure 1C**; **Supplementary Figure S1B**), viral gene expression was significantly lower in SARS-CoV-2 Omicron BA.1-infected mice compared to the other groups, and day 2 viral RNA loads were significantly higher for Delta than for the other variants (**Figure 1C**). Since the type I and III interferon (IFN) responses are the earliest host responses to be triggered upon viral infection, we next measured the gene expression of *Ifna5*, *Ifnb*, and *Ifnl* in lung tissue. For *Ifna5* gene expression, there were no significant differences, although levels of mRNA were highest at day 2 in Delta-infected mice (**Figure 1D**). Correlating with the higher viral RNA loads (**Figure 1C**), *Ifnb* and *Ifnl* gene expression was significantly higher in SARS-CoV-2 Delta-infected mice at day 2 p.i. compared to D614G-infected mice, while no *Ifn* gene expression was detected in the lungs of SARS-CoV-2 Omicron BA.1-infected mice (**Figure 1D**). To investigate downstream ISG expression, we analyzed the mRNA levels of antiviral effectors *Mx1, Oas1*, and *Viperin* in lung tissue, which were all highly increased in SARS-CoV-2 D614G and Delta-infected mice at day 2 p.i. but returned to baseline by day 7, while gene expression was not detected in SARS-CoV-2 Omicron BA.1-infected mice (**Figure 1E**). The same gene expression pattern was detected for the type I IFN-induced chemokine *Cxcl10* (**Supplementary Figure S1C**). We also assessed the gene expression of pro-inflammatory mediators *Cxcl1*, *Ccl2*, and *Il6* and found that while gene expression was highly increased in the lungs of SARS-CoV-2 D614G-infected mice, it was significantly lower in SARS-CoV-2 Delta-infected mice and not induced in SARS-CoV-2 Omicron BA.1-infected mice (**Figure 1F**).

### SARS-CoV-2 Omicron BA.1-infected WT mice and induce an ISG response

**Figure 2:**
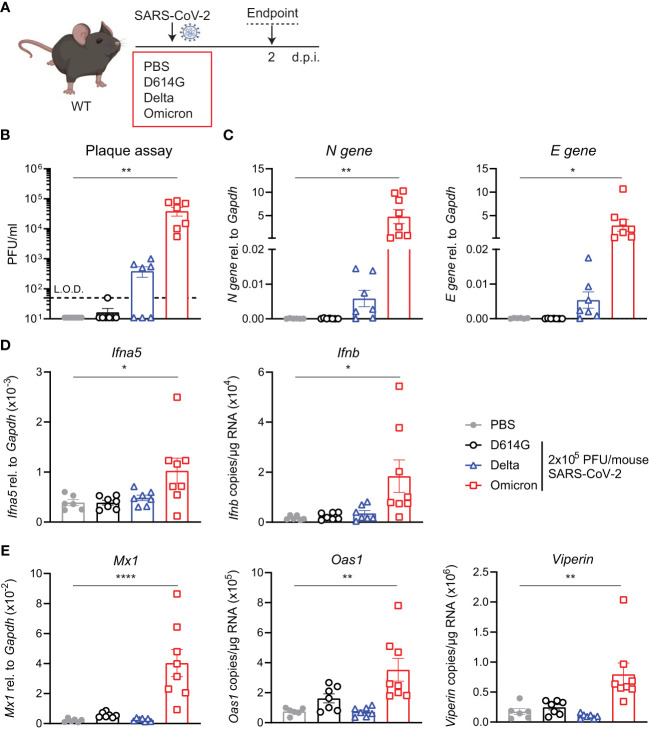
Viral load and ISG responses are higher in C57BL/6 WT mice upon SARS-CoV-2 Omicron BA.1 than D614G or Delta infection. **(A)** C57BL/6 WT mice were intranasally infected with 2 × 10^5^ PFU SARS-CoV-2 D614G, Delta or Omicron variants, or mock (PBS). Lungs and BAL were harvested at 2 days post-infection (d.p.i). **(B)** Viral load measured by plaque assay on Vero cells overexpressing hACE2 and TMPRESS2, limit of detection (L.O.D. = 50 PFU/mL). **(C)** Expression of SARS-CoV-2 *N gene* (nucleocapsid phosphoprotein) and *E gene* (envelope protein) in lung tissue relative to *Gapdh*, measured by RT-PCR. **(D)** Gene expression analysis of *Ifna5* (relative to the expression of *Gapdh*) and *Ifnb* (total copy number normalized to the expression of *Gapdh*) measured by RT-PCR. **(E)** Gene expression analysis of ISGs; *Mx1, Oas1*, and *Viperin* measured by RT-PCR, relative to the expression of *Gapdh* (*Mx1*) or total copy numbers normalized to the expression of *Gapdh* (*Oas1* and *Viperin*). Data are shown as mean ± SEM; two experiments pooled, *n* = 6–8 per group. One-way ANOVA with Dunnett’s multiple-comparison test was used. * indicates significance compared to PBS group; *p < 0.05, **p < 0.01, ****p < 0.001.

The increased binding of the Omicron spike to mouse ACE2 has been extensively reported by others and has even led to suggestions of a rodent origin for the Omicron VOC (24). We were interested to test whether such increased binding would counteract the otherwise poor replication of Omicron BA.1 in mice, as seen in the *hACE2* ^knock-in^ mice (**Figure 1**). Therefore, we next infected C57BL/6 WT mice with the same titer of the three SARS-CoV-2 variants; first-wave D614G, Delta, or Omicron, and assessed viral load and innate immune responses at day 2 p.i. This time point was chosen since this was the time point that showed the most significant differences in the previous experiment (**Figure 2A**). Infectious virus in the lungs was detected by plaque assay (**Figure 2B**) and SARS-CoV-2 N and E gene expression by RT-PCR as before (**Figure 2C**). In contrast to the virus replication patterns in the *hACE2* ^knock-in^ mice, Omicron replicated to the highest titers in WT mice, attaining titers between 10^4^ and 10^5^ PFU/mL lung extract. Furthermore, only infection with Omicron resulted in a significant induction of *Ifna5* and *Ifnb* (**Figure 2D**), ISG-driven antiviral effectors *Mx1*, *Oas1*, and *Viperin* (**Figure 2E**), and pro-inflammatory mediators *Cxcl10* and *Il6* (**Supplementary Figure S2**).

### Increased SARS-CoV-2 Omicron BA.1 infection in *Ifnar1^-/-^* mice

**Figure 3:**
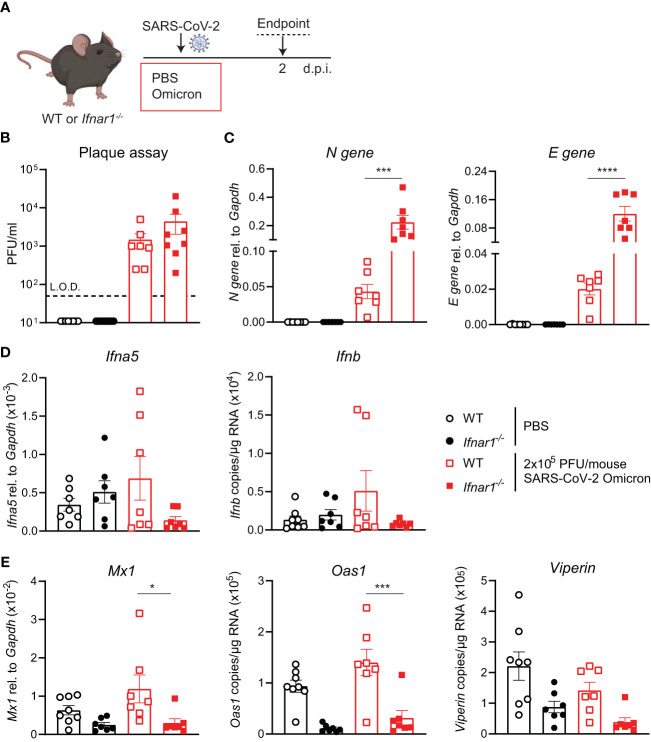
Increased viral replication in C57BL/6-*Ifnar1^-/-^* mice upon SARS-CoV-2 Omicron BA.1 infection. **(A)** C57BL/6 WT or *Ifnar1^-/-^* mice were intranasally infected with 2 × 10^5^ PFU SARS-CoV-2 Omicron (B.1.1.529) or mock (PBS). Lungs and BAL were harvested at 2 days post-infection (d.p.i). **(B)** Viral load measured by plaque assay on Vero cells overexpressing hACE2 and TMPRESS2. **(C)** Expression of SARS-CoV-2 *N gene* (nucleocapsid phosphoprotein) and *E gene* (envelope protein) in lung tissue relative to *Gapdh*, measured by RT-PCR. **(D)** Gene expression analysis of *Ifna5* (relative to the expression of *Gapdh*) and *Ifnb* (total copy number normalized to the expression of *Gapdh*) measured by RT-PCR. **(E)** Gene expression analysis of ISGs; *Mx1, Oas1*, and *Viperin* measured by RT-PCR, relative to the expression of *Gapdh* (*Mx1*) or total copy numbers normalized to the expression of *Gapdh* (*Oas1* and *Viperin*). Data are shown as mean ± SEM; two experiments pooled, *n* = 7–8 per group. One-way ANOVA with Tukey’s multiple-comparison test per time point was used. * indicates significance compared to SARS-CoV-2 WT-infected group; *p < 0.05, ***p < 0.005, ****p < 0.001.

Since SARS-CoV-2 Omicron BA.1 efficiently infected WT mice and induced innate responses (**Figure 2**), we next investigated the impact of type I IFN receptor signaling on the outcome of SARS-CoV-2 Omicron BA.1 infection. Using hACE2-AAV transduced *Ifnar1^-/-^* and WT mice infected with SARS-CoV-2 D614G, we have previously shown that type I IFN receptor signaling impairment results in higher viral loads and altered innate immune responses, with particularly increased neutrophil and Ly6C^-^ inflammatory myeloid cell recruitment to the lung (21). Here WT or *Ifnar1^-/-^* mice were infected with 2 × 10^5^ PFU SARS-CoV-2 Omicron BA.1, and lungs were harvested at day 2 p.i. as before (**Figure 3A**). While the infectious viral titers measured by plaque assay were not significantly higher in *Ifnar1^-/-^* compared to WT mice (**Figure 3B**), SARS-CoV-2 N and E gene expression was significantly increased in *Ifnar1^-/-^* mice (**Figure 3C**). The gene expression of type I IFNs—*Ifna5* and *Ifnb*—was variable and not significantly increased in WT-infected *versus* uninfected animals, although it was consistently low in *Ifnar1^-/-^* mice (**Figure 3D**). As expected in *Ifnar1^-/-^* mice, the gene expression of the antiviral ISGs—*Mx1, Oas1*, and *Viperin* (**Figure 3E**)—as well as *Cxcl10* (**Supplementary Figure S3**) was decreased compared to WT mice infected with SARS-CoV-2 Omicron BA.1. The gene expression of pro-inflammatory mediators *Cxcl1*, *Ccl2*, and *Il6* was not significantly altered in this model at day 2 p.i. with SARS-CoV-2 Omicron BA.1 in either WT or *Ifnar1^-/-^* mice (**Supplementary Figure S3**).

### SARS-CoV-2 D614G^[Omicron-spike]^ chimeric virus allows the infection of WT mice

**Figure 4:**
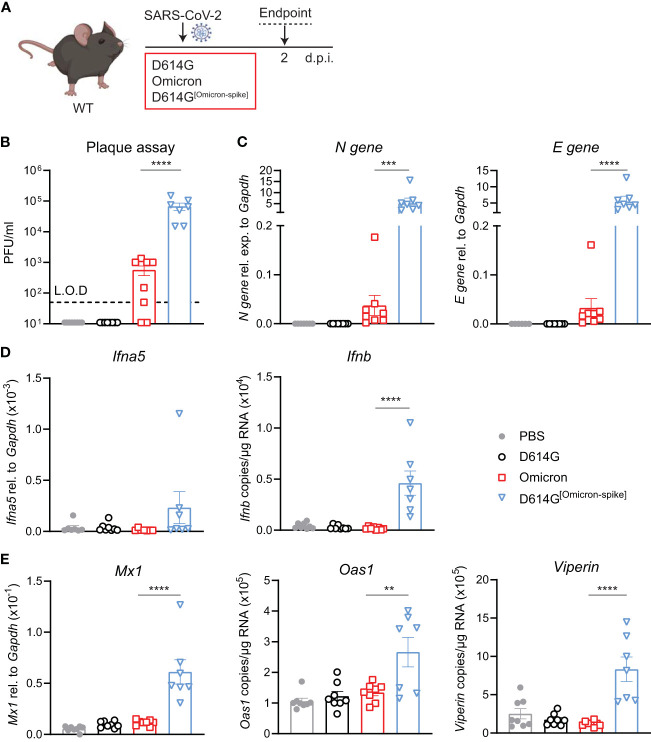
Omicron BA.1-spike expression results in cell entry but genes from SARS-CoV-2 D614G are important for viral replication and induction of innate immunity in C57BL/6 mice. **(A)** C57BL/6 WT mice were intranasally infected with 5 × 10^4^ PFU SARS-CoV-2 D614G, Omicron BA.1, D614G^[Omicron-spike]^, or mock (PBS). Lungs and BAL were harvested at 2 days post-infection (d.p.i). **(B)** Viral load measured by plaque assay on Vero cells overexpressing hACE2 and TMPRESS2, limit of detection (L.O.D. = 50 PFU/mL. **(C)** Expression of SARS-CoV-2 *N gene* (nucleocapsid phosphoprotein) and *E gene* (envelope protein) in lung tissue relative to *Gapdh*, measured by RT-PCR. **(D)** Gene expression analysis of *Ifna5* (relative to the expression of *Gapdh*) and *Ifnb* (total copy number normalized to expression of *Gapdh*) measured by RT-PCR. **(E)** Gene expression analysis of ISGs; *Mx1, Oas1*, and *Viperin* measured by RT-PCR, relative to the expression of *Gapdh* (*Mx1*) or total copy numbers normalized to the expression of *Gapdh* (*Oas1* and *Viperin*). Data are shown as mean ± SEM; two experiments pooled, *n* = 7–8 per group. One-way ANOVA with Tukey’s multiple-comparison test per time point was used; * indicates significance compared to SARS-CoV-2 Omicron BA.1-infected group; **p < 0.01, ***p < 0.005, ****p < 0.001.

To validate that the increased infection by the Omicron BA.1 in WT mice was indeed due to the mutations in the Spike protein, we used a chimeric virus in which the Omicron BA.1 S gene was substituted in the SARS -CoV-2 first-wave genome in place of homologous D614G S. WT mice were intranasally administered either 5 × 10^4^ PFU SARS-CoV-2 D614G, Omicron BA.1 or D614G^[Omicron-spike]^, or PBS, and lungs were harvested at day 2 p.i. (**Figure 4A**). Note that this infection dose was lower than that used in the experiments in the previous figures due to the lower titers of the chimeric virus stock. Viral load was detectable in the lungs of SARS-CoV-2 D614G^[Omicron spike]^- but not SARS-CoV-2 D614G-infected mice by both plaque assay (**Figure 4B**) and N and E gene expression assay (**Figure 4C**), and titers of chimeric virus were significantly increased compared to Omicron BA.1. While the gene expression of *Ifna5* was variable, *Ifnb* gene expression was consistently and significantly increased in SARS-CoV-2 D614G^[Omicron spike]^- as opposed to Omicron BA.1-infected mice (**Figure 4D**). Furthermore, SARS-CoV-2 D614G^[Omicron spike]^ infection resulted in a significantly increased expression of ISGs *Mx1, Oas1*, and *Viperin* (**Figure 4E**) as well as *Cxcl10* (**Supplementary Figure S4**), while pro-inflammatory mediators *Cxcl1, Ccl2*, and *Il6* were not significantly altered in any group compared to the uninfected mice (**Supplementary Figure S4**). These results confirm that attenuation of Omicron BA.1 in mice is at least partly mediated by non-spike genes. Combining the more efficient entry of virus bearing the Omicron BA.1 spike into cells expressing murine ACE2, with the less mouse attenuated non-spike backbone of the first-wave SARS CoV-2 virus, resulted in a more robust infection in WT mice.

## Discussion

Our experiments show that replication of SARS-CoV-2 Omicron BA.1 is attenuated in the lungs of *hACE2^knock-in^* mice compared to SARS-CoV-2 D614G and Delta. Attenuated replication of Omicron BA.1 in mice has also been reported in other model systems that offer both human and murine ACE2 for entry, such as the K18-*hACE2* model (16, 23). This suggests that other genetic determinants outside of those involved in receptor attachment contribute to Omicron BA.1’s mouse attenuation.

Although no weight loss is detected in the *hACE2^knock-in^* model, it may arguably be advantageous for comparing different VOCs since here *hACE2* displaces mouse *Ace2* (14), and different infectivity is not due to additional entry via mouse ACE2. An interesting observation from this work is that Delta infection results in the highest viral load on day 2 p.i. compared to the infection with D614G but at the same time had a lower pro-inflammatory cytokine and chemokine expression, suggesting a change in the way Delta VOC interacts with the host innate immune response (25).

The Omicron spike protein has been shown to efficiently bind to murine ACE2 as entry receptor due to residue mutations Q493R, Q498R, and N501Y (24), and there have been reports showing that it can also access cells via the endosomal route (11). Accordingly, we detected significant viral titers in the lungs of Omicron BA.1-infected WT C57BL/6 WT mice at day 2 p.i., similar to BALB/c mice (26, 27). Existing data on Omicron infection in C57BL/6 mice is variable (28, 29), most likely dependent on infection dose and investigated timepoints. Our data suggest that significant viral titers at early timepoints p.i. drive the induction of innate immune responses such as type I IFN and ISG expression. This indicates that SARS-CoV-2 Omicron BA.1 infection of C57BL/6 WT mice could be used as an infection model, which also opens the opportunity to use knockout and transgenic mice on a C57BL/6 background.

We utilized this model and infected mice lacking type I interferon receptor alpha (*Ifnar1^-/-^*) with SARS-CoV-2 Omicron BA.1 to investigate the impact of the lack of type I IFN receptor signaling in the context of the VOC, Omicron BA.1. Although we observed increased viral loads and the absence of ISG expression in *Ifnar1^-/-^* mice upon infection with SARS-CoV-2 Omicron BA.1, the overall response to infection in this model was lower compared to our previous study of SARS-CoV-2 D614G infection in hACE2-AAV-transduced WT or *Ifnar1* ^-/-^ mice (21). This may be due to several factors: firstly, the infection titer of SARS-CoV-2 Omicron BA.1 was 10-fold lower in the system used here since the stock did not yield a higher titer. Secondly, while SARS-CoV-2 Omicron RBD may bind efficiently to murine ACE2, other parts of the Omicron spike may confer attenuation of cell entry (12). Furthermore, mutations in Omicron non-spike regions of the genome, including *nsp6*, may contribute to attenuated replication in mice (30). This may also result in an altered timeline of immune responses to SARS-CoV-2 Omicron compared to first-wave or Delta virus infection, possibly explaining why no differences in *Cxcl1* or other pro-inflammatory mediators were observed.

Using a SARS-CoV-2 D614G^[Omicron spike]^ chimeric virus, we show that efficient SARS-CoV-2 infection of C57BL/6 mice is possible if the Omicron BA.1 spike gene is combined with the non-spike region of the first-wave SARS-CoV-2 genome. However, although viral loads and immune responses were higher in the SARS-CoV-2 D614G^[Omicron spike]^ **-**infected WT mice, they were still several folds lower than those seen for SARS-CoV-2 D614G or Delta VOC in *hACE2* ^knock-in^ mice. While this suggests that further attenuating features exist in the Omicron BA.1 spike outside of the RBD, a comparison with the data shown in **Figure 1** is difficult since a lower dose of infection had to be used for SARS-CoV-2 D614G^[Omicron spike]^, and inflammatory response may only be triggered when viral titers exceed a specific threshold. Further open questions include which genes drive the enhanced replication of SARS-CoV-2 D614G^[Omicron spike]^ compared to Omicron BA.1 and which Omicron BA.1 spike mutation restored binding to the murine ACE2 receptor. Additionally, it will be important to investigate the binding of the Omicron spike to murine ACE2 structurally and to investigate other cell entry routes. In a pilot study, Peacock, Brown, and Zhou et al. show that, in immortalized human lung cells (Calu-3) and primary human nasal cells, attenuated replication of Omicron BA.1 compared to Delta VOC mapped to the spike protein since recombinant viruses with varying spike proteins recapitulated the patterns of their spike donor parent (12, 31). Nonetheless, the efficient entry and replication of chimeric virus with Omicron-spike protein in C57BL/6 mice opens up the possibility to study SARS-CoV-2 infection (using genetically modified mouse models on a C57BL/6 background). Furthermore, chimeric viruses with backbones of newly arising variants and Omicron spikes can enable the investigation of core gene mutations in more detail. Considering recent evolution of SARS-CoV-2 Omicron, this system may be further improved by generating chimeras expressing newer Omicron spike variants such as BA.2 or XBB.1.

## Data availability statement

The raw data supporting the conclusions of this article will be made available upon request by the authors, without undue reservation.

## Ethics statement

The animal study was approved by Animal Welfare and Ethical Review Board (AWERB) at Imperial College London. The study was conducted in accordance with the local legislation and institutional requirements.

## Author contributions

PO: Conceptualization, Data curation, Formal analysis, Investigation, Methodology, Visualization, Writing – original draft, Writing – review & editing. MM: Writing – review & editing, Investigation. SJ: Investigation, Writing – review & editing. JZ: Methodology, Resources, Writing – review & editing. JB: Methodology, Resources, Writing – review & editing. KS: Writing – review & editing, Resources. WF: Resources, Writing – review & editing. AP: Resources, Writing – review & editing. VC: Resources, Writing – review & editing. MP: Resources, Writing – review & editing. WB: Conceptualization, Methodology, Resources, Writing – original draft, Writing – review & editing. CJ: Conceptualization, Data curation, Funding acquisition, Investigation, Project administration, Supervision, Visualization, Writing – original draft, Writing – review & editing.
