# Supplementary material for: SARS-CoV-2 strains bearing Omicron BA.1 spike replicate in C57BL/6 mice
Source: Front Immunol. 2024 Apr 29;15:1383612. doi: 10.3389/fimmu.2024.1383612 (PMC11089223; doi:10.3389/fimmu.2024.1383612)
Supplement: Supplementary file 1 [file DataSheet_1.pdf]

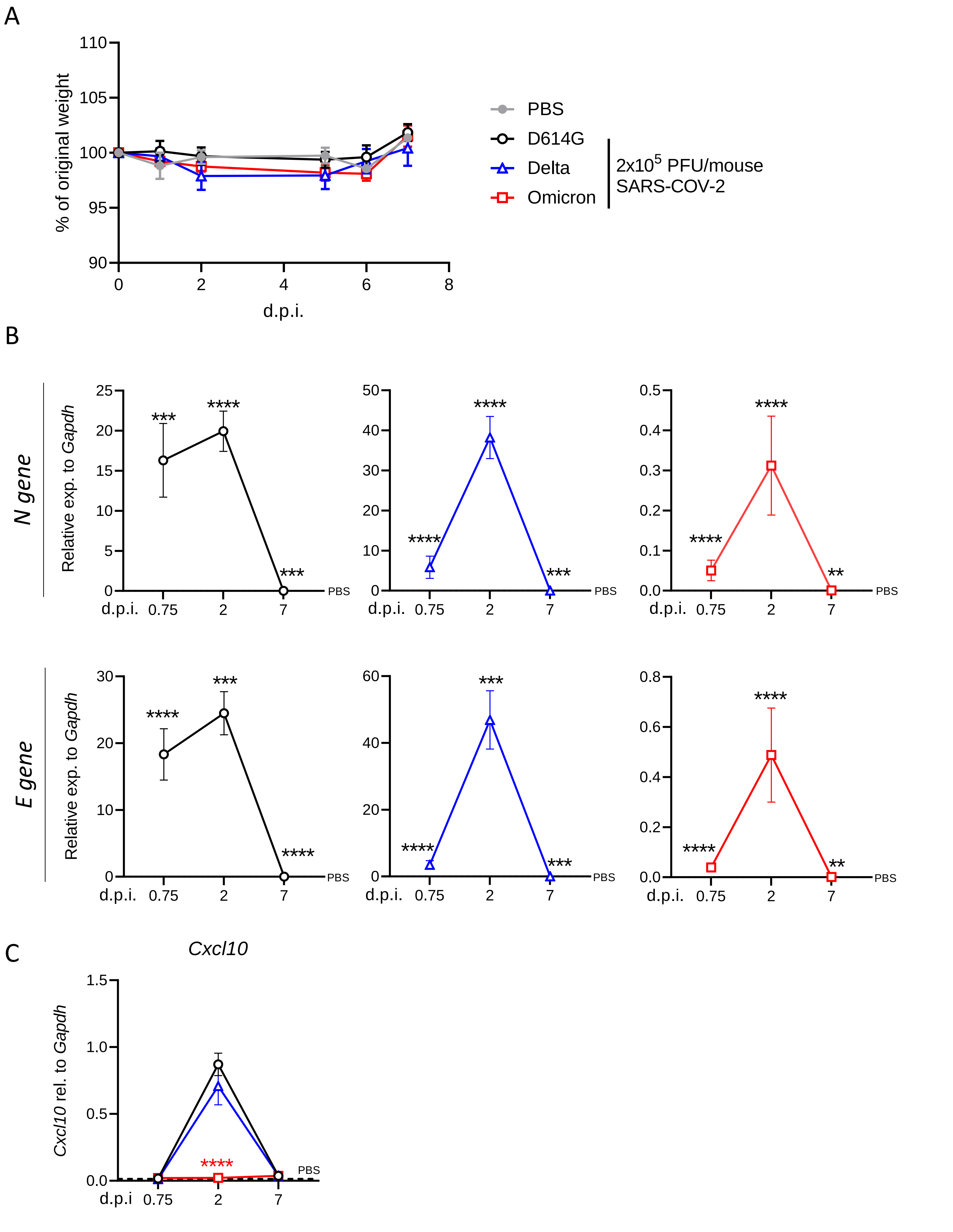

Figure S1: Viral load in *hACE2*$^{knock\text{-}in}$ mice infected with SARS-CoV-2 D614G, Delta and Omicron

A) Weight loss up to 7 d.p.i. in *hACE2*$^{knock\text{-}in}$ mice post infection with SARS-CoV-2 **B)** Expression of SARS-CoV-2 *N gene* (nucleocapsid phosphoprotein) and *E gene* (envelope protein) in lung tissue relative to *Gapdh*, measured by RT-PCR * indicates significant compared to PBS control group**.** **C)** Gene expression of chemokine *Cxcl10* measured by RT-PCR relative to expression of *Gapdh.* Data are shown as mean ± SEM; 2 experiments pooled, n = 6 - 8 per group, PBS control n = 11, shown as dotted line**.** One Way ANOVA + Tukey's multiple comparison test per time point; * P < 0.05, ** P < 0.01, *** p < 0.005, **** p < 0.001.

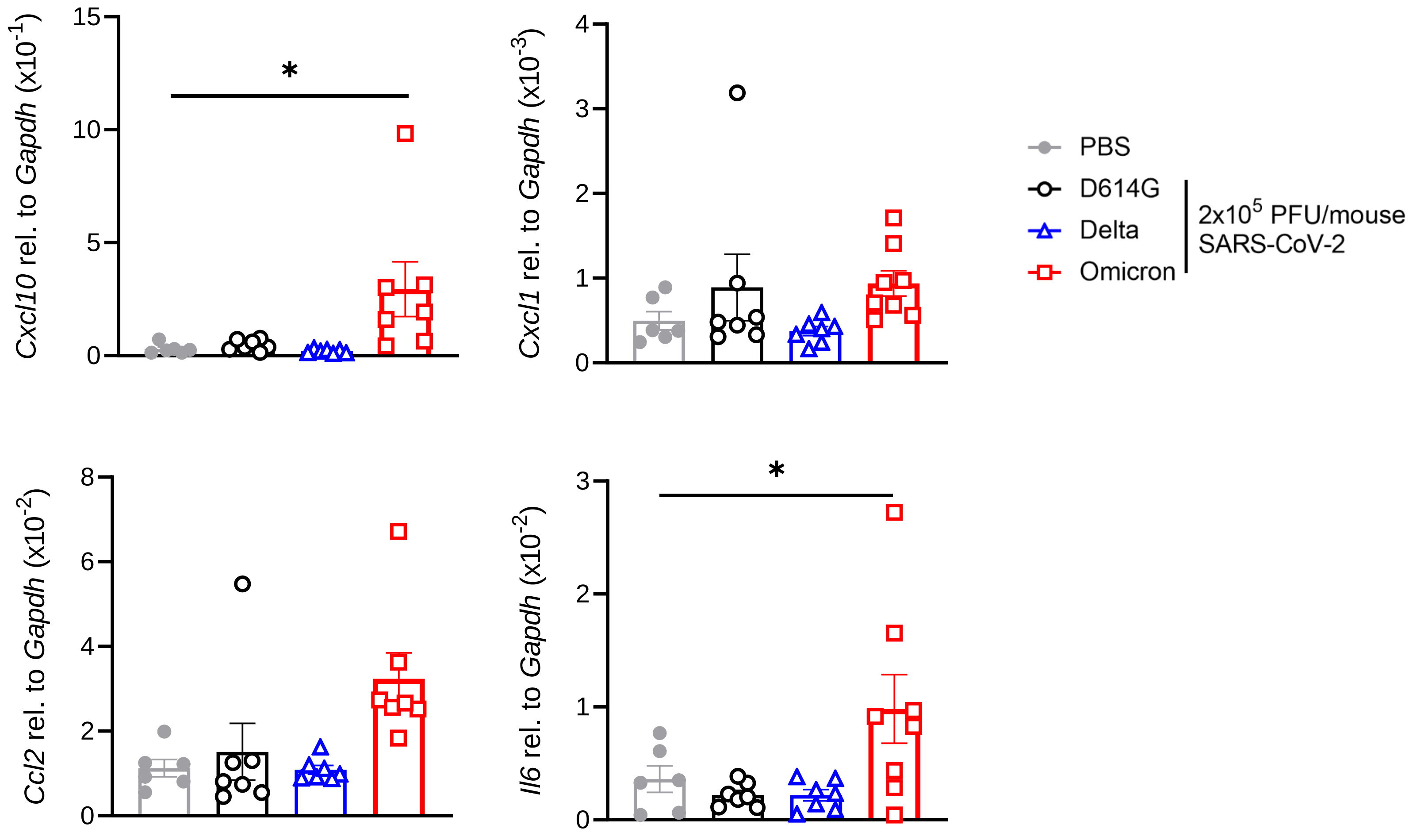

Figure S2: Analysis of cytokine and chemokine gene expression in C57BL/6 WT mice upon infection with various SARS-CoV-2 strains

Gene expression of chemokines *Cxcl10, Cxcl1, Ccl2* and cytokine *Il6*, measured by RT-PCR relative to expression of *Gapdh* in lung tissue. Data are shown as mean ± SEM; 2 experiments pooled, n = 6 - 8 per group. One Way ANOVA + Tukey's multiple comparison test per time point; * $P < 0.05$.

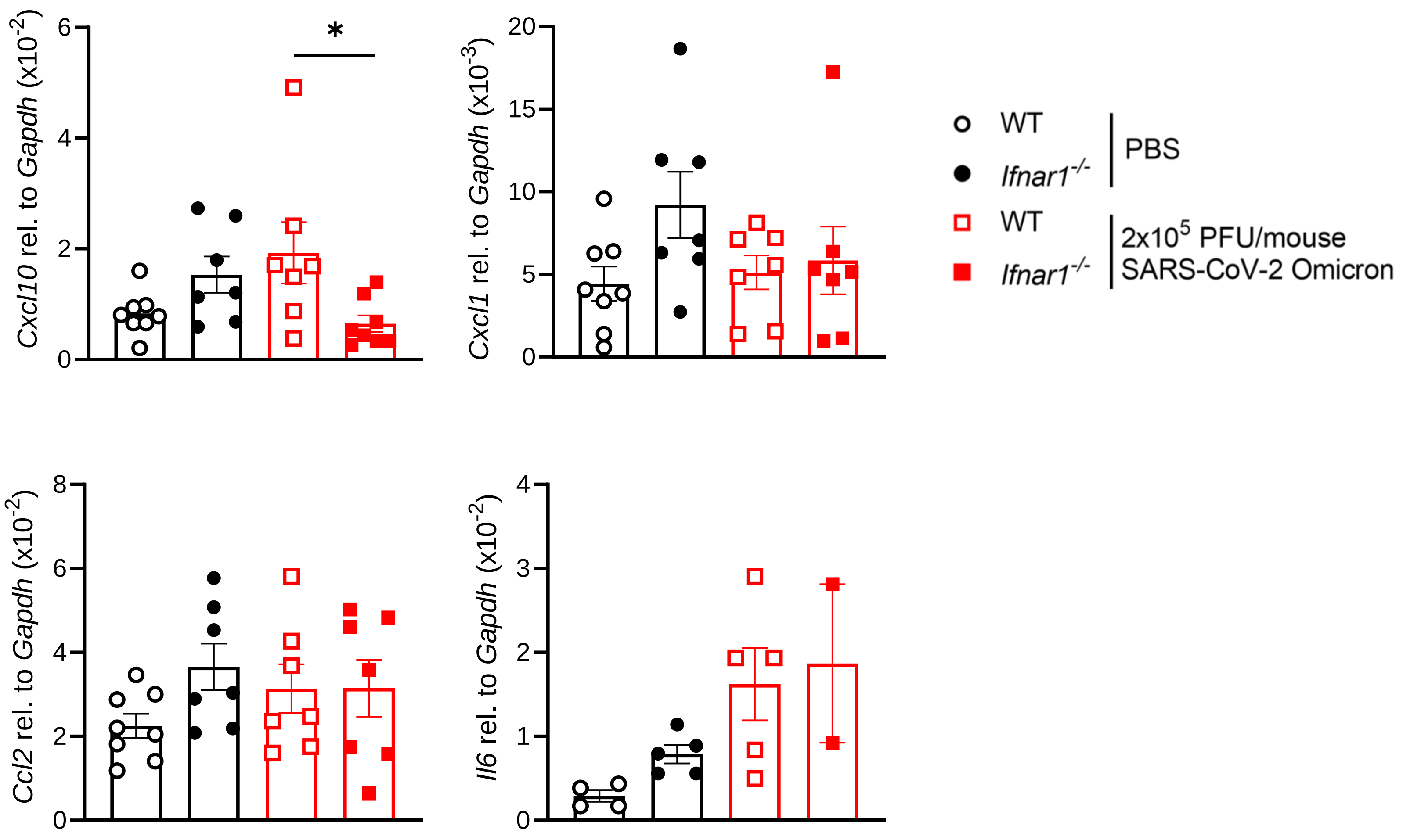

Figure S3: Analysis of cytokine and chemokine gene expression in C57BL/6 WT and $Ifnar^{-/-}$ mice infected with SARS-CoV-2 Omicron

Gene expression of chemokines *Cxcl10, Cxcl1, Ccl2,* and cytokine *Il6*, measured by RT-PCR relative to expression of *Gapdh* in lung tissue*.* Data are shown as mean ± SEM; 2 experiments pooled, n = 6 - 8 per group (*Il6* only 1 experiment). One Way ANOVA + Tukey's multiple comparison test per time point; * P < 0.05.

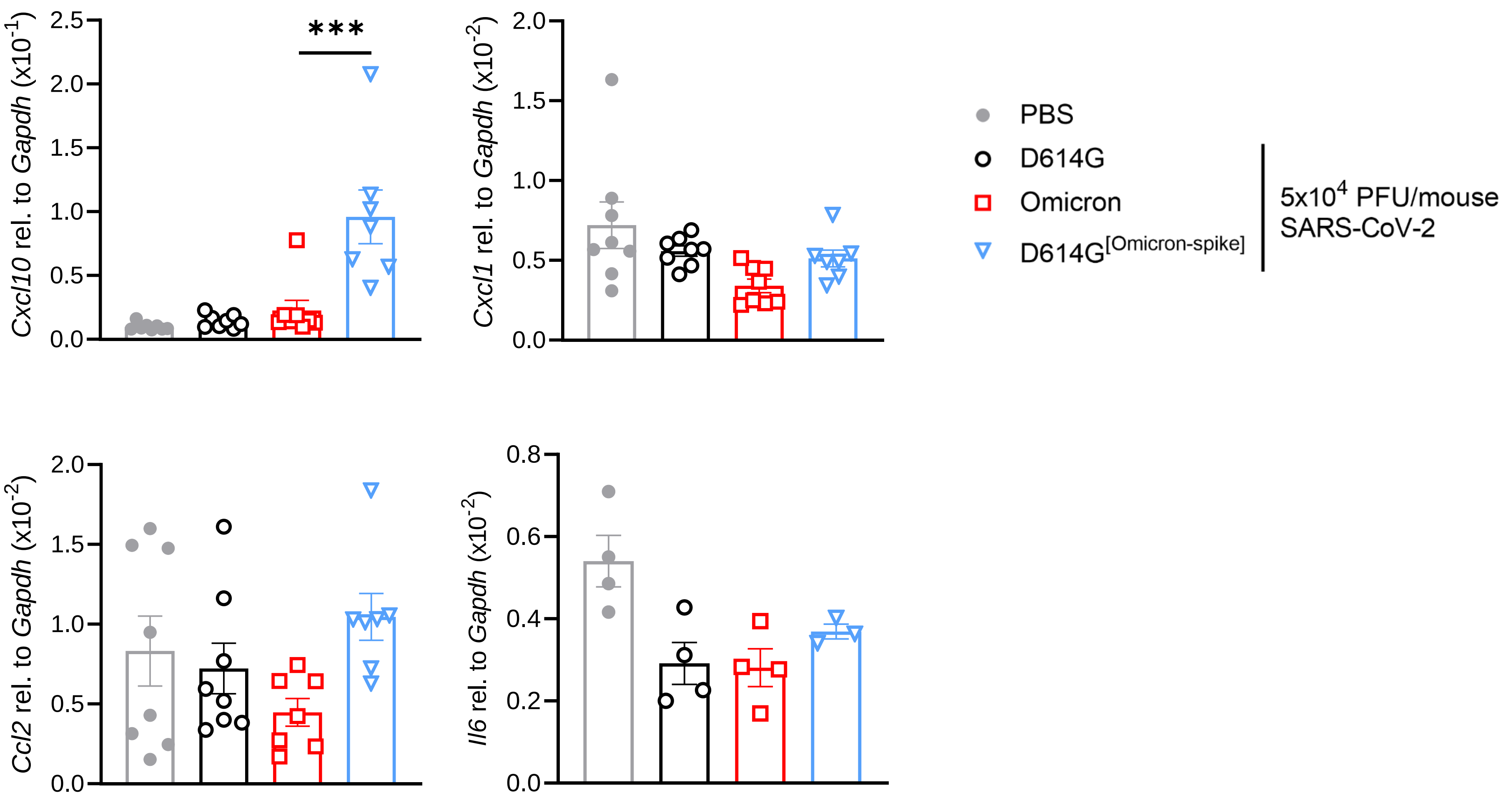

Figure S4: Analysis of cytokine and chemokine gene expression in C57BL/6 WT mice infected with Omicron or D614G[Omicron-spike]

Gene expression of chemokines *Cxcl10, Cxcl1, Ccl2,* and cytokine *Il6*, measured by RT-PCR relative to expression of *Gapdh* in lung tissue. Data are shown as mean ± SEM; 2 experiments pooled, n = 6 - 8 per group (*Il6* only 1 experiment). One Way ANOVA + Tukey's multiple comparison test per time point; *** $p < 0.005$.
